# A Bad Habit

**Published:** 1875-02-15

**Authors:** Charles Hervey Hunt

**Affiliations:** Stanwood, Iowa


### A BAD HABIT.

BY CHARLES HERVEY HUNT, M.D., STANWOOD, IOWA.

IT is my wish in a brief article to call attention to a habit which is very prevalent, and about which much might be said. The habit to which I refer is that of sitting with the feet elevated. I am not aware that any medical writer has ever touched upon this subject and the symptoms produced by it. Any one who will take the trouble to observe and examine for themselves, will be convinced that it is very common, more especially among business men, students, and those who lead an inactive life. Nature never intended for us to keep our feet above our heads, or she would have put them there. The first thought may be that "there is nothing in it," but if one will practice it with a view of finding out the truth for themselves, they will be convinced that it ought to be called a bad habit. Should you call upon a business man at his office and engage him in conversation, the chances are that he will have his feet upon the desk. The same thing happens while he is reading his morning paper, or doing anything else that does not require his feet to be on the floor. The habit becomes so fixed that whenever he sits down he is uneasy until he is in position. No matter where he is, the disposition to get the heels higher than the head manifests itself.

My attention was called to the habit for the first time when a student, while boring my way into the mysteries of physic. My favorite position was to sit with my feet upon the top of a desk, or a chair, which brought them upon a level with my head. After pursuing this course for about three months, I began to be troubled with headache, a feeling of lassitude, and a sense of oppression in the cardiac region, with palpitations of the heart now and then. The cardiac region seemed very sensitive and every beat of the heart could be felt. Occasionally it would cease and then start up with one great thump, as it does when one receives a sudden fright. Not knowing the cause of these symptoms,

I began to labor under the impression that I was developing some serious disease. Being new in the study of disease, my reading on that subject did not tend to diminish that idea. Soon I was obliged to throw down my books and take to outdoor exercise. For a week I pursued this course, and to my surprise the symptoms had nearly vanished. Shortly after this I went to lectures. Here the old position was resumed, the seats being admirably arranged for that purpose. In a month the trouble returned worse than before, and I was obliged to take to my bed for a couple of days. Still believing that a serious disease of the heart was rapidly developing itself, I had one of the professors of the college call, and after making an examination, he said, much to my relief, that I was "all right." Determined to find the cause of the trouble, I began to search around for it. One day the idea occurred to me that sitting with the feet up might be the cause. To prove this I tried it for a week, when the same symptoms began to appear again. After this I kept my feet on the floor, where they belonged, for a couple of weeks. Gradually the symptoms ceased, not to return again, for I have tried to keep in "position" since.

The question may be asked, "How does this position cause these symptoms?"

It will be seen that if the lower extremities are flexed upon the abdomen, the femoral artery which supplies them, and which escapes under Poupart's ligament, will be compressed, and the large amount of blood which should go to these parts is prevented from doing so. Having no other outlet here, it must be thrown into the other organs of the body, viz.: the liver, spleen, kidneys, lungs and brain. The vessels become distended, and the damming up of the blood causes the heart to act with more force. The contents of the abdomen are forced upward also, encroaching upon the space occupied by the lungs and heart. The heart is forced out of its natural position, the apex being raised and carried to the left. The pulse in the wrist becomes stronger and quicker.

I have often questioned persons whom I have found in this position, and have met with but few cases where these symptoms were not found. That this habit has a bad effect upon those who have any organic disease of the heart I have no doubt, as I have found it to be true in a number of cases. Corpulent persons are more affected by this habit than lean ones, but in all it is a habit to be avoided.

---

REPORTING THE EPIDEMIC DISEASES.—The Board of Health of Philadelphia, have passed the following:—

*Resolved*, that the Health Officer be informed, that Medical Reports of all cases of the following diseases, or of any other diseases which may at any time hereafter assume, either generally or in some particular locality, a pestilential character, would meet the requirements of the Board, in the matter of pestilential or contagious diseases, viz: Asiatic Cholera, Relapsing Fever, Yellow Fever, Typhus or Ship Fever, Cerebro-spinal Meningitis or Spotted Fever, Small-pox and Varioloid, Scarlet Fever and Diphtheria. —*Phil. Med. and Surg. Rep.*

